# Neurotoxicity of Synthetic Cannabinoid Receptor Agonist Cumyl-PINACA: An In Vitro Study on Rat Cortical Neurons and Astrocytes

**DOI:** 10.3390/ph19091436

**Published:** 2026-09-10

**Authors:** Damijana Mojca Jurič, Klara Bulc Rozman, Metoda Lipnik-Štangelj, Dušan Šuput, Miran Brvar

**Affiliations:** 1 Institute of Pharmacology and Experimental Toxicology, Faculty of Medicine, University of Ljubljana, Korytkova 2, 1000 Ljubljana, Slovenia; damijana-mojca.juric@mf.uni-lj.si (D.M.J.); metoda.lipnik-stangelj@mf.uni-lj.si (M.L.-Š.); 2 Institute of Pathophysiology, Faculty of Medicine, University of Ljubljana, Zaloška cesta 4, 1000 Ljubljana, Slovenia; klara.bulc@rozman.si (K.B.R.); dusan.suput@mf.uni-lj.si (D.Š.); 3 Centre for Clinical Toxicology and Pharmacology, University Medical Centre Ljubljana, Zaloška cesta 7, 1000 Ljubljana, Slovenia; 4 Centre for Clinical Physiology, Faculty of Medicine, University of Ljubljana, Zaloška cesta 4, 1000 Ljubljana, Slovenia

**Keywords:** synthetic cannabinoid receptor agonists (SCRAs), cumyl-PINACA (SGT-24), neurons, astrocytes, neurotoxicity, receptors

## Abstract

**Background/Objectives:** Synthetic cannabinoid receptor agonists (SCRAs) are associated with severe neurotoxicity, but the cellular mechanisms underlying their effects remain poorly defined. We investigated the effects of Cumyl-PINACA (SGT-24), a carboxamide-type SCRA derived from cumylamine, on rat cortical neurons and astrocytes. **Methods:** Primary rat cortical neurons and astrocytes were exposed to 1–10,000 nM SGT-24. Metabolic activity, mitochondrial function, morphology, and cell death were evaluated. Selective antagonists of cannabinoid receptor type 1 (CB1), G protein-coupled receptor 55 (GPR55), peroxisome proliferator-activated receptor gamma (PPARγ), and transient receptor potential cation channel subfamily V member 1 (TRPV1) were used to probe the involvement of these receptor pathways. **Results:** SGT-24 decreased metabolic activity in both cell types in a concentration- and time-dependent manner, with greater potency in neurons (IC_50_ = 13.2 nM) than in astrocytes (IC_50_ = 39.8 nM). After 24 h, maximal effects were observed at 100 nM in neurons and 500 nM in astrocytes, reducing metabolic activity by 45.2% and 36.2%, respectively. At these concentrations, mitochondrial membrane potential decreased to 57.6% and 54.1% of control, while cellular ATP levels fell to 51.6% and 52.5%, respectively. Neurons predominantly exhibited early apoptosis (21.9% of cells vs. 3.1% in controls), whereas astrocytes showed mainly 7-aminoactinomycin D (7-AAD)-positive cell death (19.3% vs. 8.2% in controls). Pharmacological inhibition of CB1, TRPV1, and PPARγ attenuated SGT-24-induced metabolic impairment, mitochondrial dysfunction, and apoptosis in neurons, whereas inhibition of CB1 and PPARγ reduced astrocytic toxicity. **Conclusions**: SGT-24 exerts potent, cell-type-dependent neuroglial toxicity associated with mitochondrial dysfunction and distinct cell death patterns, suggesting the involvement of cannabinoid receptor-dependent and non-cannabinoid signalling mechanisms. The nanomolar potency of SGT-24 underscores the toxicological risk posed by high-potency SCRAs.

## 1. Introduction

Synthetic cannabinoid receptor agonists (SCRAs) were initially developed for biomedical research and showed promise as potential therapeutic agents [1]. However, they now represent the largest group of new psychoactive substances [2]. Widely available and frequently abused as cannabis substitutes, SCRAs pose a significant public health risk, with numerous reports of severe clinical adverse effects and associated fatalities [3,4,5]. Common acute effects include agitation, confusion, hallucinations, drowsiness, tachycardia, hypertension, chest pain, and vomiting [3]. Compared with cannabis, SCRAs are more often associated with severe complications, including seizures, delirium, respiratory depression, arrhythmias, myocardial infarction, stroke, and liver and kidney injury [3,5]. Chronic exposure can lead to persistent impairments in cognition, memory, behaviour, and sleep, and may increase the risk of psychiatric disorders by disrupting of the endogenous cannabinoid system [6,7,8,9].

SCRAs are highly potent full agonists at cannabinoid type 1 and type 2 receptors (CB1 and CB2, respectively), exhibiting robust cannabimimetic activity [8]. The endocannabinoid system is increasingly conceptualised as the broader endocannabinoidome, which includes additional lipid mediators, receptors, and metabolic enzymes [10]. Only a limited number of SCRAs have been investigated for interactions with non-cannabinoid targets, and most show low affinity for major neurotransmitter receptors [3,11]. Therefore, differences between SCRAs and Δ^9^-tetrahydrocannabinol (THC) are largely attributed to their greater potency and efficacy at CB1 receptors, although contributions from non-cannabinoid targets and distinct downstream signalling pathways cannot be excluded [3,11].

**Figure 1:**
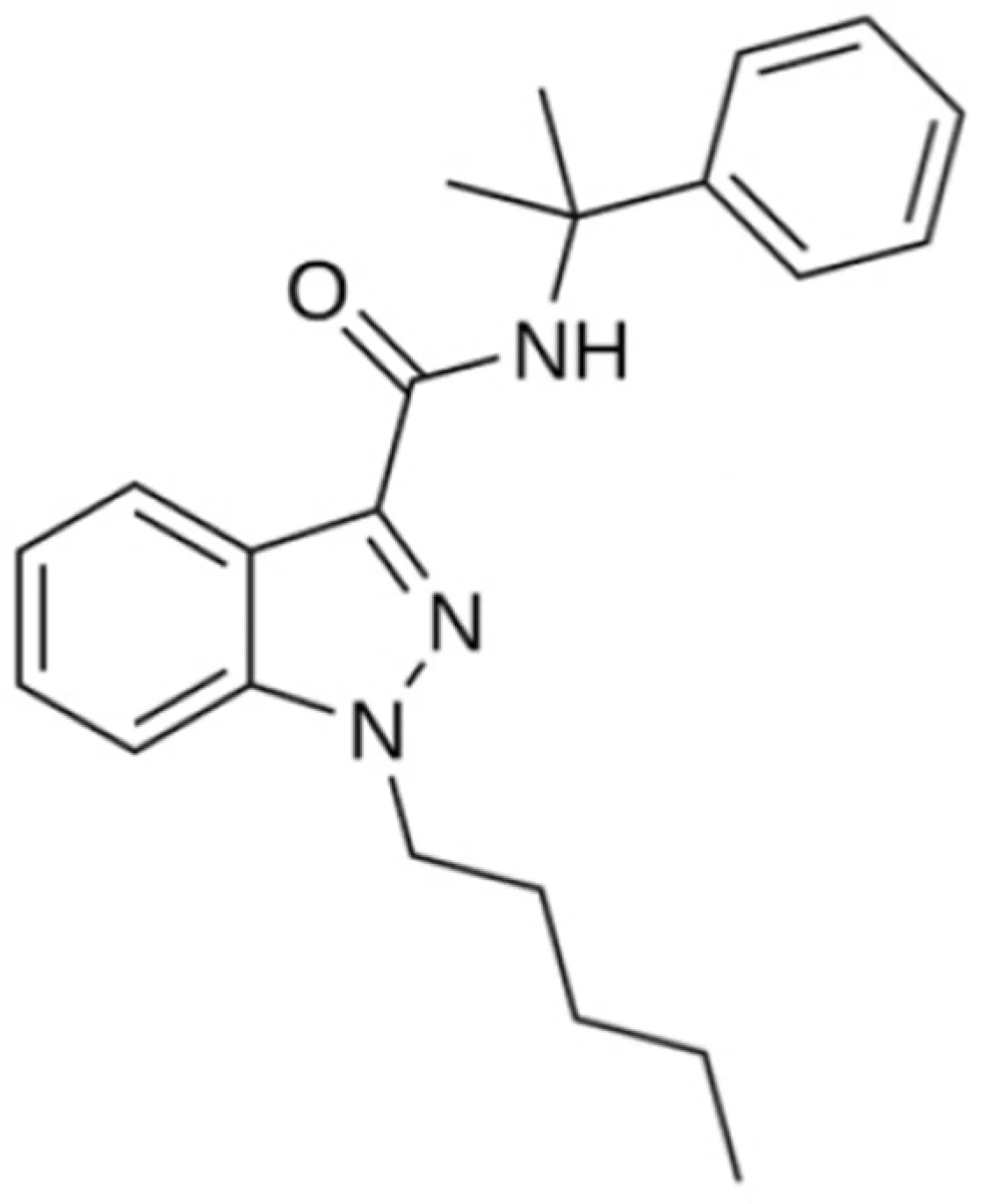
Cumyl-PINACA (SGT-24).

Cumyl-PINACA (1-pentyl-N-(2-phenylpropan-2-yl)-1H-indazole-3-carboxamide; SGT-24) (Figure 1) was originally developed for therapeutic purposes, including pain relief and appetite stimulation [12]. It is a highly potent full agonist at human CB1 and CB2 receptors, with half-maximal effective concentration (EC_50_) values of 0.15 nM and 0.41 nM, respectively, and preferential binding to CB1 receptors [13,14,15,16]. Its clinical relevance became evident after accidental occupational transdermal exposure to concentrated Cumyl-PINACA caused severe and prolonged cannabinoid intoxication [17]. Patients presented with somnolence, confusion, mydriasis, ataxia, tachycardia, and orthostatic hypotension, with symptoms persisting for up to two days. Pharmacokinetic studies demonstrated prolonged systemic presence, with detection in serum for up to 17 h and metabolites persisting in urine for more than eight days [15,18].

Consistent with its high central nervous system multiparameter optimisation (CNS MPO) score, Cumyl-PINACA demonstrates strong brain penetration potential [16]. Despite its potent pharmacological profile, the molecular mechanisms underlying its neurotoxic effects are poorly characterised. In this study, we investigated the cellular mechanisms underlying the effects of acute exposure to Cumyl-PINACA on rat cortical neurons and astrocytes.

## 2. Results

### 2.1. Presence of Cannabinoid-Related Receptors

Pharmacological evidence indicates that cannabinoids modulate signalling through both canonical cannabinoid receptors (CBRs) and non-canonical targets [19,20,21], activating multiple receptors within a single cell, located on the plasma membrane and in the nucleus [22]. Among these, G protein-coupled receptor 55 (GPR55), the ionotropic transient receptor potential vanilloid 1 (TRPV1) channel, and the nuclear peroxisome proliferator-activated receptor gamma (PPARγ)—all implicated in endocannabinoid signalling [23,24,25,26]—may, alongside CB1 and CB2, contribute to the cytotoxic effects of SGT-24 [21].

**Figure 2:**
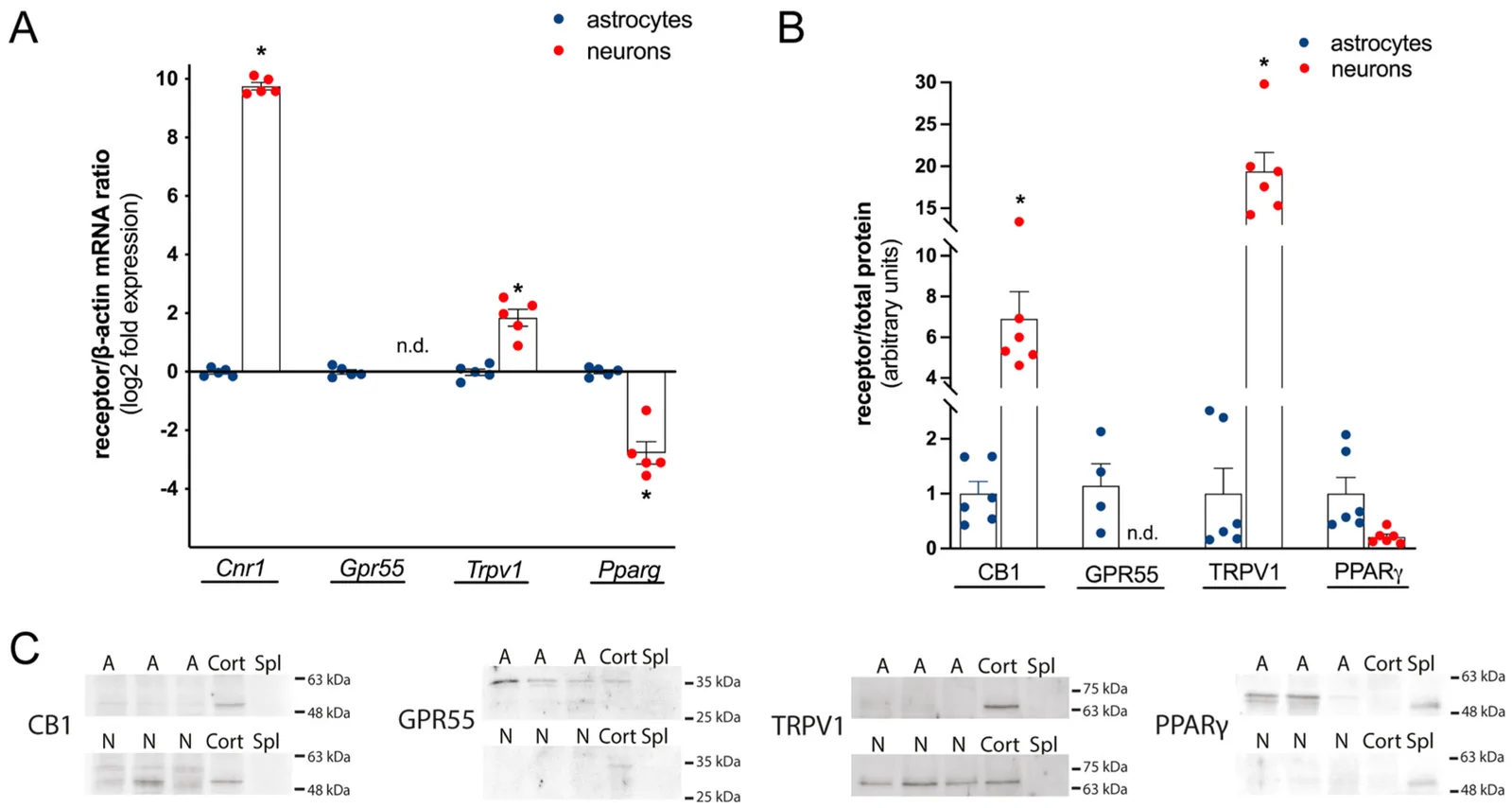
Expression of cannabinoid receptor type 1 (CB1; *Cnr1*), G protein-coupled receptor 55 (GPR55; *Gpr55*), transient receptor potential cation channel subfamily V member 1 (TRPV1; *Trpv1*), and peroxisome proliferator-activated receptor gamma (PPARγ; *Pparg*) in cultured rat cortical neurons and astrocytes. (**A**) mRNA expression levels were determined in neurons and astrocytes using quantitative real-time PCR. Gene expression was normalised to *ß-actin*, which served as an internal control. For each gene, expression levels in neurons are presented as fold changes relative to astrocytes, which were assigned a value of 1.0. Data are presented as mean ± SEM (*n* = 5). Statistical significance was assessed using an independent-samples *t*-test. Significant differences in receptor mRNA expression between neurons and astrocytes are indicated by * *p* < 0.05. n.d., not detected. (**B**,**C**) Protein expression levels were determined in neurons (N) and astrocytes (A) by Western blotting. Samples of rat brain cortices (Cort) and spleen (Spl) served as positive or negative antibody controls, respectively. Protein expression levels were normalised to astrocytes and are presented as mean ± SEM (*n* = 4–6). Statistical significance was assessed using Student’s *t*-test. Significant differences in receptor protein expression between neurons and astrocytes are indicated by * *p* < 0.05. n.d., not detected. (**C**) Representative immunoblots for each receptor are shown.

We assessed the mRNA and protein expression levels of these receptors in cultured rat cortical neurons and astrocytes using RT-PCR (Figure 2A) and Western blotting (Figure 2B,C). All receptors, except CB2, were detected at varying levels in both cell types. In contrast to some previous reports [27] and in agreement with a study on rat astrocytes [28], CB2 receptor expression was below the detection limit in both assays, indicating insufficient abundance for reliable detection under our experimental conditions. GPR55 was detected in astrocytes but was below the detection limit in cultured neurons at both the mRNA and protein levels. This finding is consistent with reports showing predominant *Gpr55* mRNA in primary mouse and rat cortical astrocytes compared with neurons [29]. The observed cell type-specific receptor expression pattern (Figure 2) is consistent with the dynamic, context-dependent distribution of cannabinoid-related receptors in the central nervous system [21,22].

### 2.2. SGT-24 Decreases Metabolic Activity

**Figure 3:**
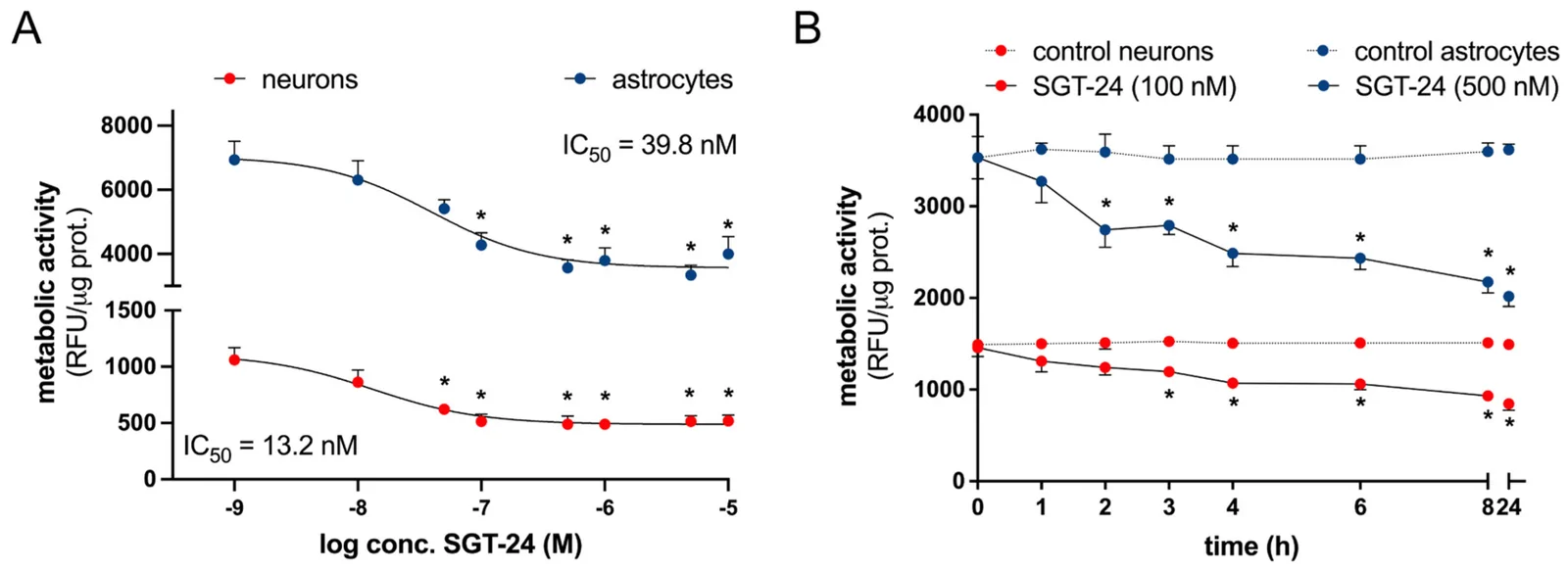
SGT-24 affects metabolic activity in cultured rat cortical neurons and astrocytes in a concentration- and time-dependent manner. (**A**) Cells were exposed to increasing concentrations of SGT-24 (1 nM–10 μM) for 24 h. Cellular metabolic activity was assessed using the AlamarBlue^®^ assay. Data are shown as relative fluorescence units (RFU), normalised to protein content, and presented as mean ± SEM (*n* = 5). Half-maximal inhibitory concentration (IC_50_) values were estimated by nonlinear regression. Statistical analysis was performed using one-way ANOVA followed by Dunnett’s post hoc test. * *p* < 0.05 vs. control. (**B**) Neurons were exposed to 100 nM SGT-24 and astrocytes to 500 nM SGT-24 for 1–24 h. Cellular metabolic activity was assessed using the AlamarBlue^®^ assay. Data are shown as relative fluorescence units (RFU), normalised to protein content, and presented as mean ± SEM (*n* = 5). Statistical analysis was performed using one-way ANOVA followed by Dunnett’s post hoc test. * *p* < 0.05 vs. control.

Primary rat cortical neurons and astrocytes were exposed to SGT-24 (1 nM–10 μM) or vehicle control, and metabolic activity was assessed after 24 h using a resazurin-based AlamarBlue^®^ assay. SGT-24 reduced metabolic activity in a concentration-dependent manner in both cell types, with neurons being more sensitive than astrocytes, as reflected by lower IC_50_ values (13.2 nM and 39.7 nM, respectively; Figure 3A). In neurons, the effect became significant at 50 nM and reached a plateau at 100 nM, with no further decrease at higher concentrations. In astrocytes, the response was less pronounced and plateaued at concentrations above 500 nM (Figure 3A).

To examine the time course of this effect, neurons and astrocytes were exposed to concentrations corresponding to the plateau phase of the concentration-response curves (100 nM and 500 nM, respectively) for 1–24 h (Figure 3B). In astrocytes, metabolic activity decreased by 23.6% after 2 h, whereas a similar reduction was observed in neurons after 3 h, despite the fivefold lower SGT-24 concentration. After 24 h, SGT-24 reduced metabolic activity by 45.2% in neurons and 36.2% in astrocytes (Figure 3B).

### 2.3. SGT-24 Impairs Mitochondrial Function

**Figure 4:**
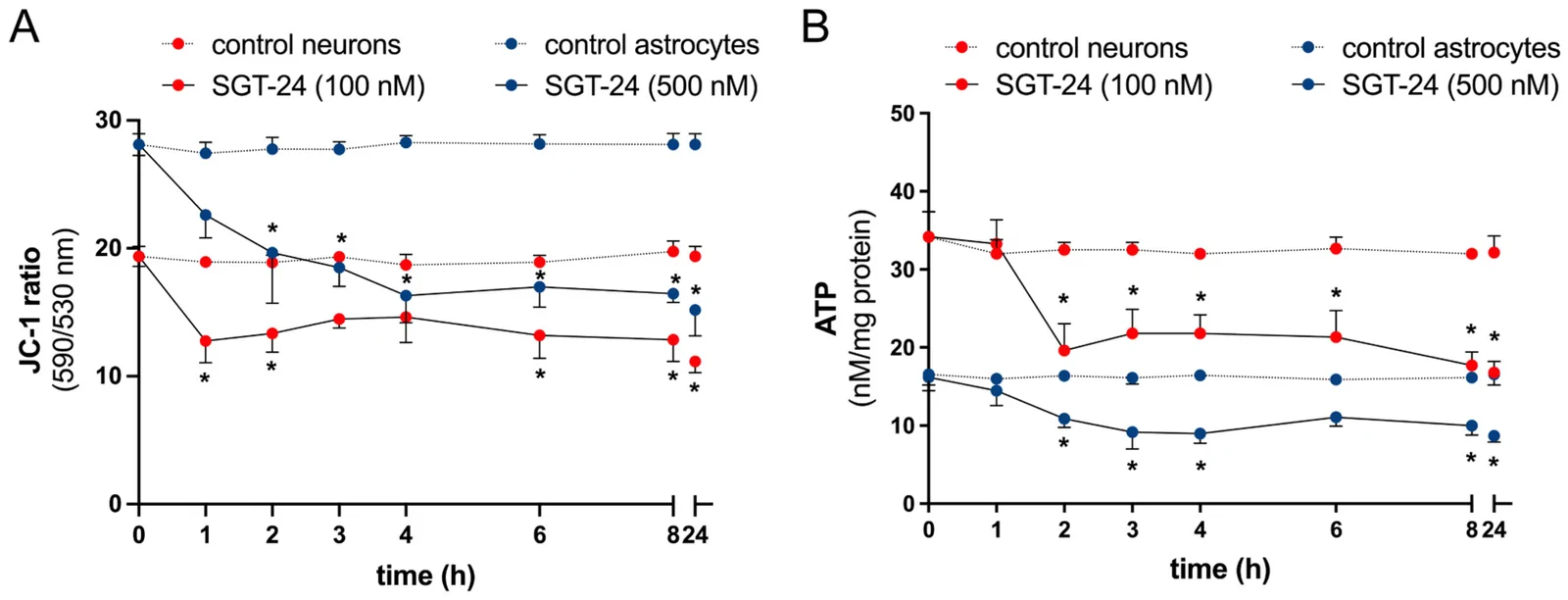
SGT-24 induces mitochondrial dysfunction in cultured rat cortical neurons and astrocytes. Neurons were exposed to 100 nM SGT-24 and astrocytes to 500 nM SGT-24 for 1 to 24 h. (**A**) Mitochondrial membrane potential (ΔΨm) was assessed using the JC-1 dye. Data (JC-1 fluorescence ratio) are presented as mean ± SEM (*n* = 5). Statistical analysis was performed using one-way ANOVA followed by Dunnett’s post hoc test. * *p* < 0.05 vs. control. (**B**) Cellular ATP content was determined using the ATPlite 1-step assay system and normalised to total protein concentration. Basal ATP content was 32.2 ± 2.1 nM/mg cell protein in cortical neurons and 16.6 ± 1.3 nM/mg cell protein in astrocytes. Results are presented as mean ± SEM (*n* = 5). Statistical analysis was performed using one-way ANOVA followed by Dunnett’s post hoc test. * *p* < 0.05 vs. control.

Exposure of cultured rat cortical cells to SGT-24 significantly impaired mitochondrial function, as shown by a rapid collapse of the mitochondrial inner membrane potential (ΔΨm) (Figure 4A). Following an initial phase of partial recovery in neurons, but not in astrocytes, ΔΨm progressively declined with continued exposure, reaching 57.6% and 53.9% of control levels in neurons and astrocytes, respectively.

To determine whether the loss of ΔΨm was accompanied by impaired cellular bioenergetics, intracellular ATP levels were measured (Figure 4B). SGT-24 exposure reduced ATP levels to 60.3% of control in neurons after 2 h and to 54.7% of control in astrocytes after 4 h. Although ATP levels showed a transient partial recovery in both cell types, prolonged exposure to SGT-24 caused a further decline, reaching 51.6% and 52.5% of control levels in neurons and astrocytes, respectively.

### 2.4. SGT-24 Induces Distinct Cell Death Responses

**Figure 5:**
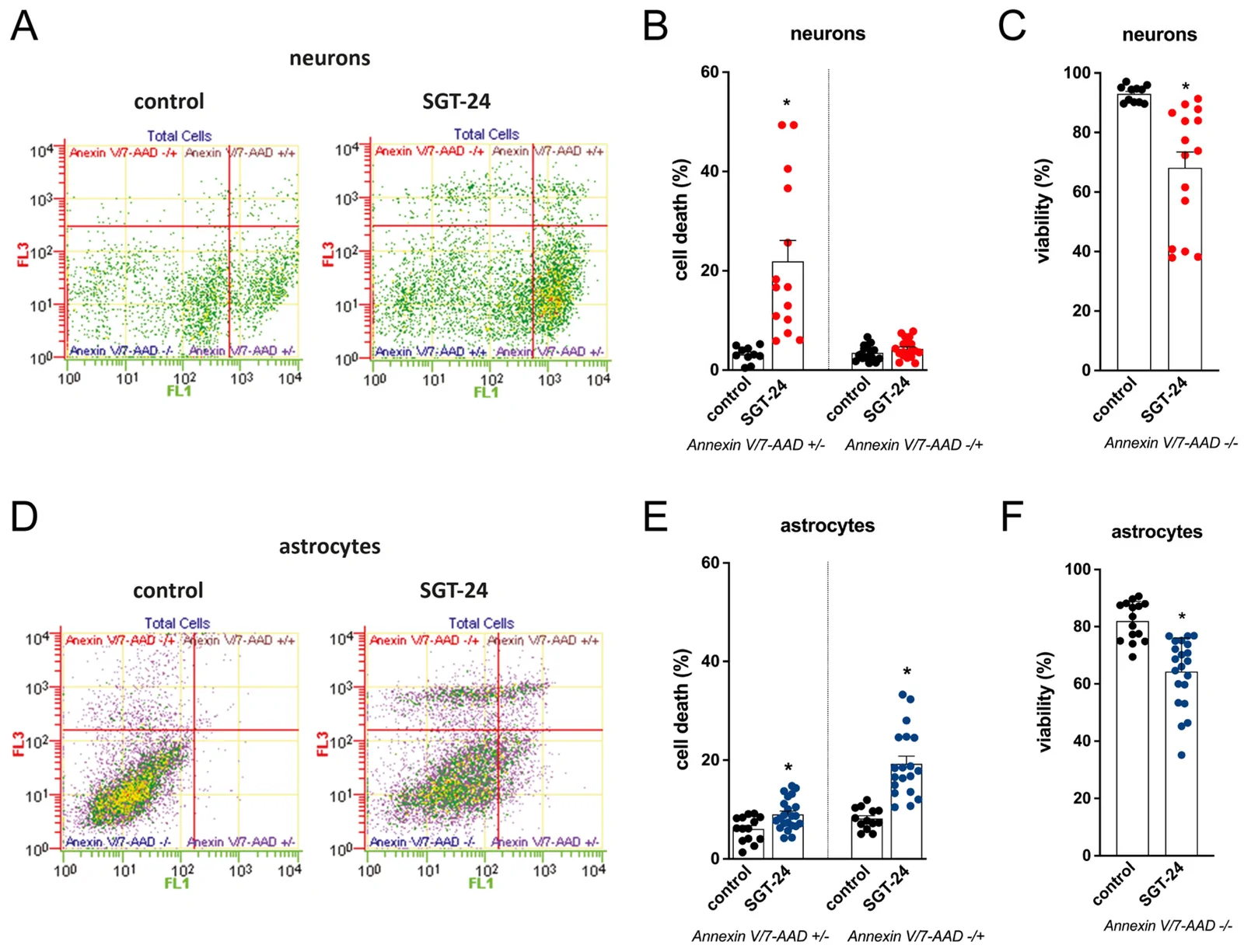
SGT-24 reduces the viability of rat cortical neurons and astrocytes by inducing distinct forms of cell death. Flow cytometry measurements were performed on neurons incubated with 100 nM SGT-24 and astrocytes incubated with 500 nM SGT-24 for 24 h, followed by a 22 h regeneration period. (**A**) Representative flow cytometry dot plot images showing Annexin V (FL1) binding and 7-AAD (FL3) uptake in neurons after treatment with 100 nM SGT-24. Control cells were not exposed to SGT-24. (**B**) Neuronal survival after 24 h exposure to 100 nM SGT-24 and 22 h regeneration, showing percentages of Annexin V/7-AAD +/− and Annexin V/7-AAD −/+ cells. Data are presented as mean ± SEM. Independent-samples *t*-test, * *p* < 0.05 vs. control. (**C**) Neuronal viability determined by flow cytometry, revealing percentages of overall cell viability (Annexin V/7-AAD −/−). Data are presented as mean ± SEM. Independent-samples *t*-test, * *p* < 0.05 vs. control. (**D**) Representative flow cytometry dot plot images showing Annexin V (FL1) binding and 7-AAD (FL3) uptake in astrocytes after treatment with 500 nM SGT-24. Control cells were not exposed to SGT-24. (**E**) Astrocyte survival after 24 h exposure to 500 nM SGT-24 and 22 h regeneration, showing percentages of Annexin V/7-AAD +/− and Annexin V/7-AAD −/+ cells. Data are presented as mean ± SEM. Independent-samples *t*-test, * *p* < 0.05 vs. control. (**F**) Astrocyte viability determined by flow cytometry, revealing percentages of overall cell viability (Annexin V/7-AAD −/−). Data are presented as mean ± SEM. Independent-samples *t*-test, * *p* < 0.05 vs. control.

**Figure 6:**
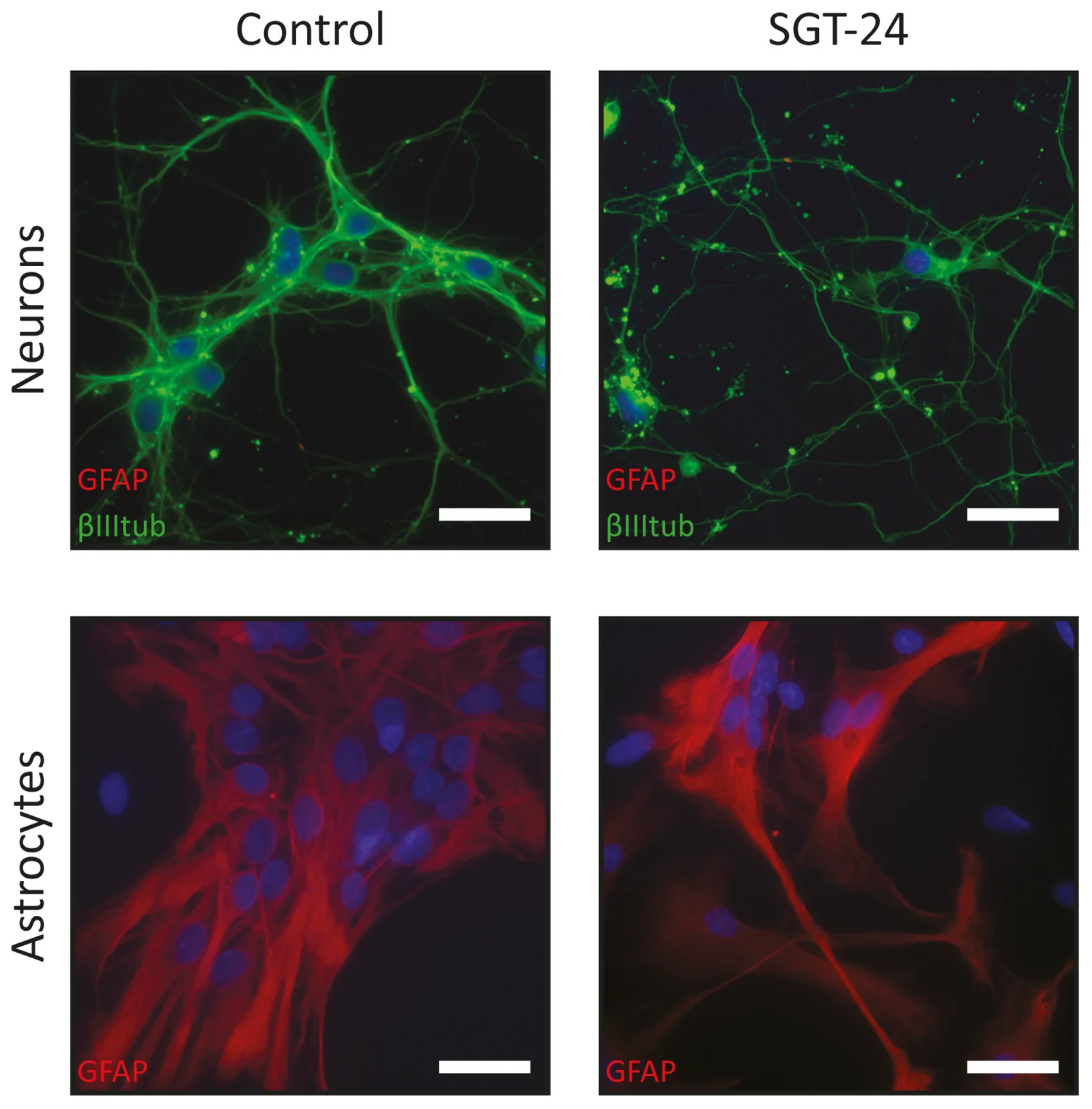
SGT-24 alters cell morphology. Representative fluorescence images of cultures after 24 h incubation with vehicle (control) or SGT-24. Neurons were exposed to 100 nM SGT-24, and astrocytes to 500 nM SGT-24. Cultures were stained for neuron-specific β-III-tubulin (green), astrocyte-specific GFAP (red), and nuclei (blue). Scale bar: 30 µm.

**Figure 7:**
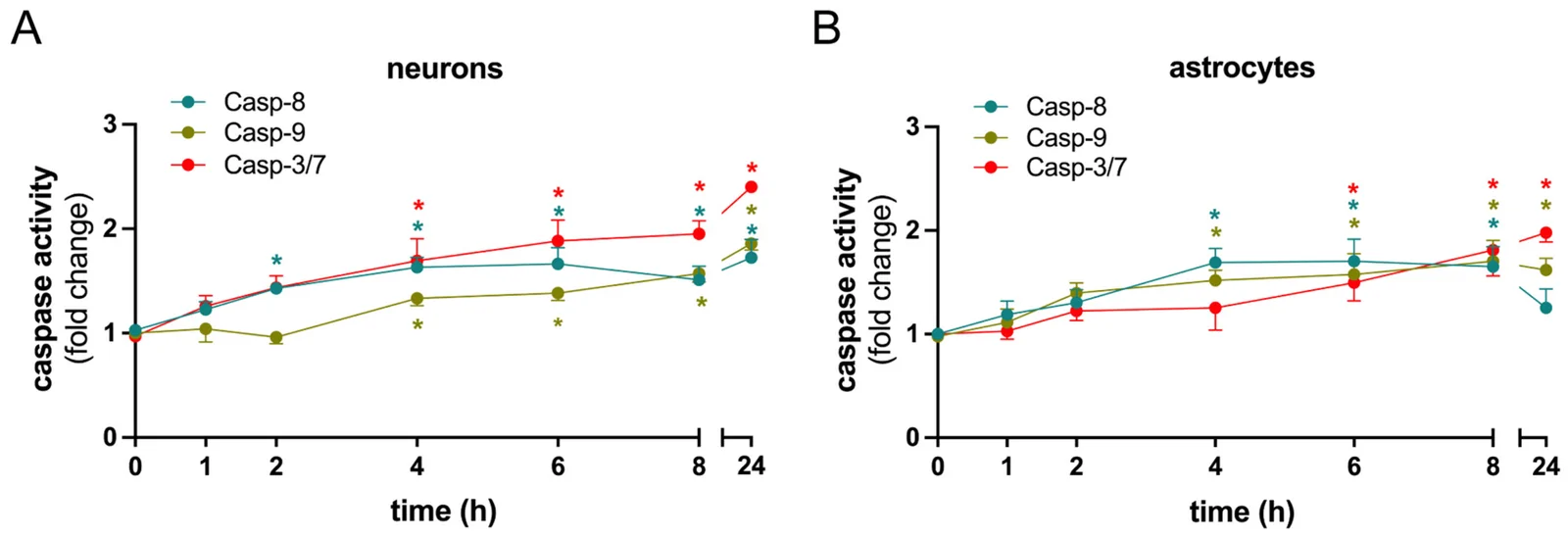
SGT-24-induced caspase activation in cultured rat cortical neurons and astrocytes. (**A**) Neurons were exposed to 100 nM SGT-24 for 1 to 24 h, and (**B**) astrocytes were exposed to 500 nM SGT-24 for the same duration. Caspase activity was evaluated using Caspase-Glo 8, Caspase-Glo 9, and Caspase-Glo 3/7 assays; data were normalised to the mean value of the control and presented as mean ± SEM (*n* = 5). Statistical analysis was performed using one-way ANOVA with Dunnett’s post hoc test. * *p* < 0.05 vs. control.

As a decrease in ΔΨm is an early event in regulated cell death [30], we investigated the mechanisms underlying the SGT-24-induced reduction in neural cell viability. Flow cytometric analysis (Figure 5), together with morphological assessment (Figure 6) and caspase activation profiling (Figure 7), revealed distinct cell death responses in neurons and astrocytes. To distinguish between different death modalities, Annexin V/7-AAD staining was used to identify healthy cells (Annexin V/7-AAD −/−), cells undergoing early apoptosis (Annexin V/7-AAD +/−), late apoptotic and/or secondary necrotic cells (Annexin V/7-AAD +/+) and cells undergoing alternative forms of cell death (Annexin V/7-AAD −/+).

Once the cell plasma membrane is perforated, Annexin V can easily enter the cell and give a positive result; therefore, it is not clear whether a given cell has died by apoptosis or necrosis. In addition, it has been shown that the binding of Annexin V precedes the formation of membrane ruptures and necrosis. This observation suggests that secondary necrotic cells (Annexin V/7-AAD+/+) do not necessarily die by apoptosis. Therefore, to avoid potential bias, cells with an Annexin V/7-AAD−/+ phenotype were considered to die via a non-apoptotic pathway, while secondary necrotic cells were omitted from the analyses (Figure 5).

Following 24 h exposure to 100 nM SGT-24, cortical neurons exhibited a pronounced increase in early apoptotic cells (21.9 ± 0.7% compared to 3.1 ± 0.4% in controls), whereas the Annexin V/7-AAD −/+ population remained unchanged (Figure 5B). SGT-24 treatment reduced neuronal viability by 24.8 ± 0.8% (Figure 5C). In astrocytes, exposure to 500 nM SGT-24 caused only a modest increase in early apoptotic cells (9.0 ± 0.6% vs. 6.1 ± 0.7% in controls; Figure 5E), whereas the Annexin V/7-AAD −/+ population increased markedly (19.3 ± 1.6% vs. 8.2 ± 0.6% in controls). This was accompanied by a 17.7 ± 0.8% reduction in cell viability (Figure 5F).

Morphological analysis revealed cell type-specific responses to SGT-24. Neurons displayed dendritic retraction, cell shrinkage, and chromatin condensation (Figure 6), features characteristic of apoptosis [31]. In contrast, astrocytes exhibited reduced cell density, cell shrinkage, cytoplasmic vacuolation, and collapse of the GFAP filament network towards the nucleus (Figure 6), consistent with severe cellular stress and structural disruption.

The observed mitochondrial dysfunction was associated with activation of apoptotic signalling pathways. In cortical neurons, SGT-24 induced early activation of caspase-8, followed by activation of caspase-9 and executioner caspase-3 (Figure 7A). Together with the Annexin V/7-AAD staining and morphological changes, these findings indicate that SGT-24 predominantly triggers caspase-dependent apoptosis in neurons. In astrocytes, caspase-8 and caspase-9 were activated concurrently, followed by activation of caspase-3/7 (Figure 7B). Caspase-8 activity declined by 24 h, whereas caspase-3 activity persisted and preceded the accumulation of Annexin V/7-AAD−/+ cells, suggesting a cell death phenotype in astrocytes that combines apoptotic signalling with progressive membrane-compromised cell death.

### 2.5. SGT-24 Acts Through the Canonical CB1 Receptor and Non-CB1 Targets

To determine whether the toxic effects of SGT-24 are mediated by CB1 receptor activation and to assess the potential contribution of non-CB1 targets, we performed pharmacological inhibition studies using selective receptor antagonists.

**Figure 8:**
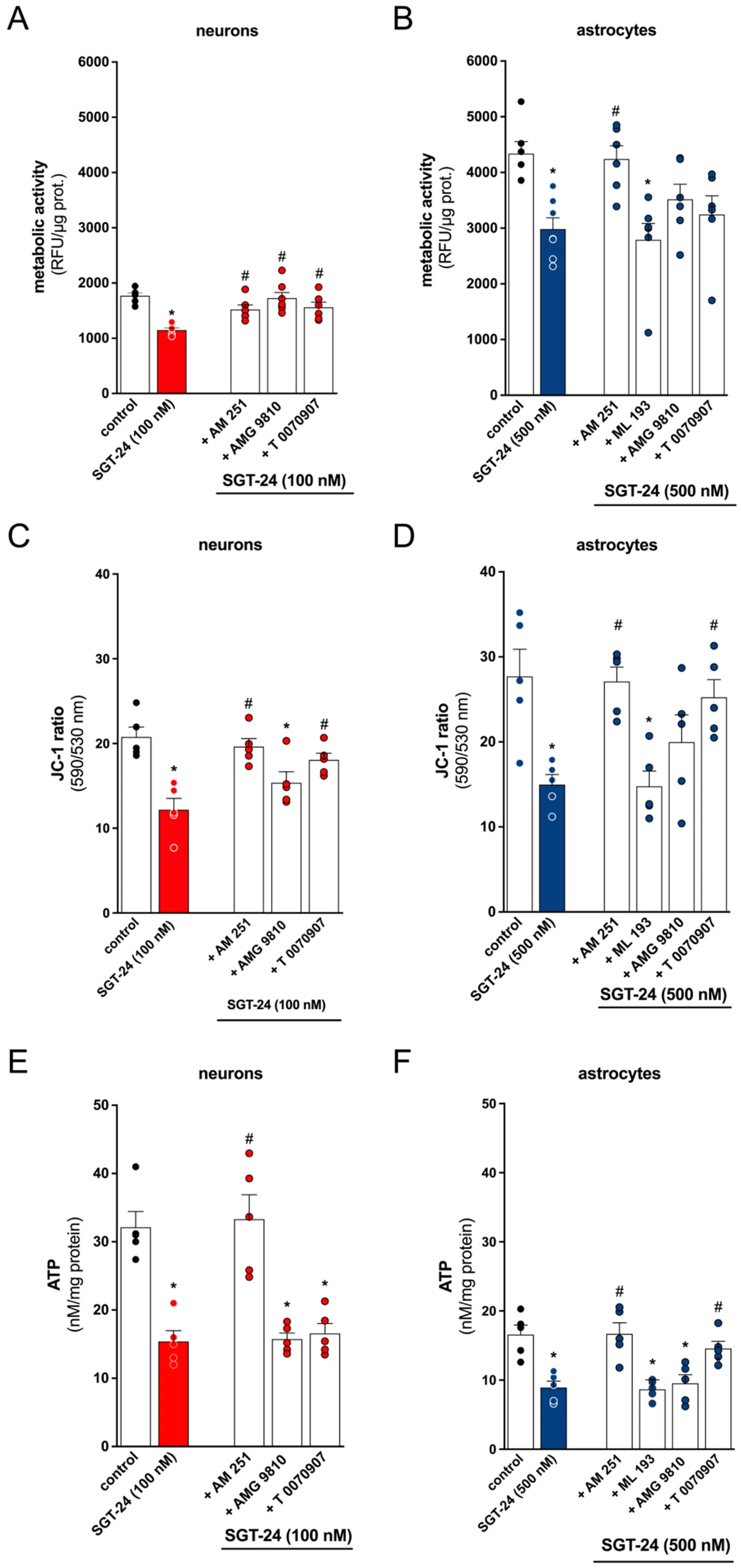
SGT-24-induced mitochondrial dysfunction in cultured rat cortical neurons and astrocytes is attenuated by CB1 and non-CB1 targets. Neurons were preincubated for 1 h with selective antagonists of CB1 (AM 251), TRPV1 (AMG 9810), or PPARγ (T 0070907) (1 μM) before a 24-h challenge with SGT-24 (100 nM) and astrocytes were preincubated for 1 h with specific antagonists of CB1 (AM 251), GPR55 (ML 193), TRPV1 (AMG 9810), or PPARγ (T 0070907) (1 μM) before a 24-h challenge with SGT-24 (500 nM). (**A**,**B**) Cellular metabolic activity was assessed using the AlamarBlue^®^ assay. Data are shown as relative fluorescence units (RFU), normalised to protein content, and presented as mean ± SEM (*n* = 6). Statistical analysis was performed using one-way ANOVA followed by Tukey’s post hoc test. * *p* < 0.05 vs. control; # *p* < 0.05 vs. SGT-24. (**C**,**D**) Mitochondrial membrane potential (ΔΨm) was assessed using the JC-1 dye. Data (JC-1 fluorescence ratio) are presented as mean ± SEM (*n* = 5). Statistical analysis was performed using one-way ANOVA followed by Tukey’s post hoc test. * *p* < 0.05 vs. control, # *p* < 0.05 vs. SGT-24. (**E**,**F**) Cellular ATP content was determined using the ATPlite 1-step assay system and normalised to total protein concentration. Results are presented as mean ± SEM (*n* = 5). Statistical analysis was performed using one-way ANOVA followed by Tukey’s post hoc test. * *p* < 0.05 vs. control; # *p* < 0.05 vs. SGT-24.

The detrimental effects of SGT-24 were partially attenuated by the selective, cell-permeable CB1 antagonist AM251 (1 µM), which significantly protected against reductions in metabolic activity (Figure 8A,B), ΔΨm disruption (Figure 8C,D), and ATP depletion (Figure 8E,F) in both neurons and astrocytes over 24 h. AM251 alone did not affect basal cellular health parameters (Supplementary Figure S1), supporting the involvement of CB1 receptor signalling in SGT-24-induced cellular toxicity.

To investigate the potential involvement of GPR55, which was detected exclusively in astrocytes, cells were treated with the selective GPR55 antagonist ML193 (1 μM). ML193 did not protect astrocytes against SGT-24-induced reductions in metabolic activity, ΔΨm disruption, or ATP depletion (Figure 8B,D,F), and had no detectable effect on basal astrocyte health parameters when applied alone (Supplementary Figure S1). These findings are consistent with those of Kevin et al. [32], who reported no measurable activity of Cumyl-PINACA at human GPR55 in a recombinant GPCR β-arrestin assay.

Given the limited information on SCRA activity at non-GPCR targets, we next investigated the possible contribution of TRPV1- and PPARγ-related pathways to SGT-24-induced toxicity. In neurons, the selective TRPV1 antagonist AMG 9810 and the PPARγ antagonist T0070907 (1 μM each) partially attenuated the reduction in metabolic activity (Figure 8A), while PPARγ inhibition also partially reduced ΔΨm disruption (Figure 8C). However, neither antagonist restored ATP levels following 24 h of SGT-24 exposure (Figure 8E). In astrocytes, PPARγ inhibition partially attenuated mitochondrial dysfunction (Figure 8D), whereas TRPV1 antagonism had no detectable effect on SGT-24-induced bioenergetic impairment (Figure 8D,F). Neither antagonist improved astrocyte metabolic activity (Figure 8B). When applied alone, neither AMG 9810 nor T0070907 produced detectable changes in basal metabolic activity, mitochondrial membrane potential, or ATP levels in either cell type (Supplementary Figure S1).

**Figure 9:**
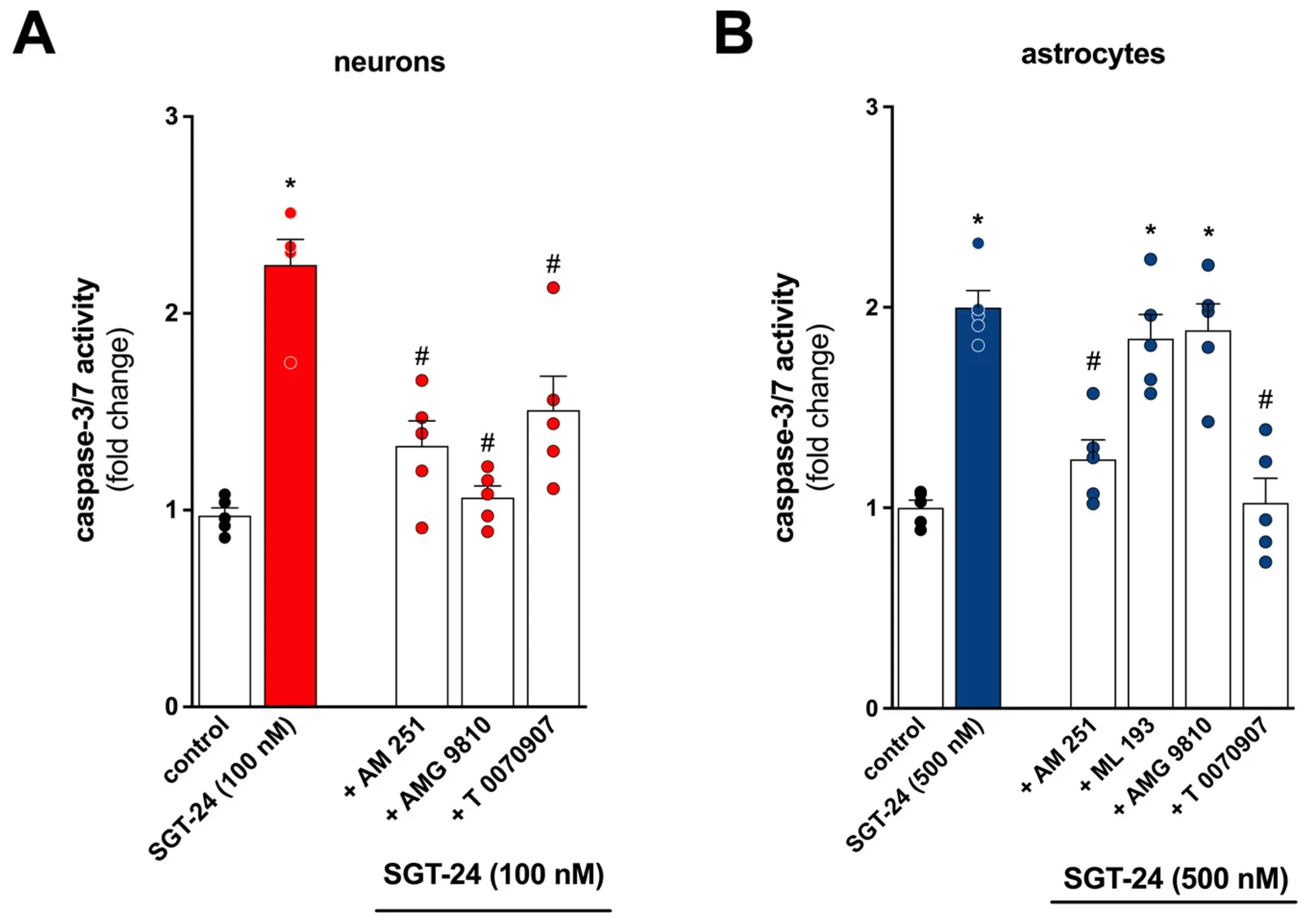
SGT-24-induced caspase 3/7 activation in cultured rat cortical neurons and astrocytes is attenuated by CB1 and non-CB1 targets. (**A**) Neurons were preincubated for 1 h with selective antagonists of CB1 (AM 251), TRPV1 (AMG 9810), or PPARγ (T 0070907) (1 μM) before a 24-h challenge with SGT-24 (100 nM) and (**B**) astrocytes were preincubated for 1 h with selective antagonists of CB1 (AM 251), GPR55 (ML 193), TRPV1 (AMG 9810), or PPARγ (T 0070907) (1 μM) before a 24-h challenge with SGT-24 (500 nM). Caspase activity was evaluated using Caspase-Glo 3/7 assays; data were normalised to the mean value of the control and presented as mean ± SEM (*n* = 5). Statistical analysis was performed using one-way ANOVA with Tukey’s post hoc test. * *p* < 0.05 vs. control; # *p* < 0.05 vs. SGT-24.

Pharmacological analysis supports the possible involvement of multiple cannabinoid-related targets in SGT-24-induced cytotoxicity. In neurons, pretreatment with AM251, T0070907, and AMG 9810 (1 μM each) significantly reduced caspase-3/7 activation and subsequent cell death (Figure 9A), suggesting that SGT-24 toxicity involves CB1 and additional non-CB1 pathways. In astrocytes, cell death was significantly reduced in the presence of CB1 and PPARγ antagonists (Figure 9B), whereas TRPV1 inhibition with AMG 9810 did not confer protection against SGT-24-induced cytotoxicity. Consistent with the metabolic and mitochondrial findings, the selective GPR55 antagonist ML193 (1 μM) did not attenuate SGT-24-induced caspase-3/7 activation in astrocytes (Figure 9B). When applied alone, none of the antagonists affected basal caspase-3/7 activity in either cell type (Supplementary Figure S2).

## 3. Discussion

SCRAs were developed to mimic the effects of THC but typically show greater potency and efficacy at cannabinoid receptors, and are therefore associated with a higher toxicological burden [8]. In this study, we investigated the cellular effects of Cumyl-PINACA (SGT-24), a carboxamide-type SCRA derived from cumylamine, in cultured rat cortical neurons and astrocytes.

Most studies have assessed SCRA cytotoxicity at high micromolar concentrations [33,34,35,36,37,38]. As SCRAs generally bind cannabinoid receptors with nanomolar affinity, concentrations above 1 μM may exceed those required for substantial cannabinoid receptor activation and may increase the likelihood of off-target effects [8,32,39,40]. SGT-24 is a high-affinity, potent full CB1 receptor agonist, with EC_50_ values in the low nanomolar range (0.06–2.3 nM, depending on the assay system) [12,13,15,16]. Consistent with this profile, SGT-24 reduced metabolic activity at nanomolar concentrations in our study, with neuronal effects evident at 50 nM and maximal at 100 nM, whereas astrocytic responses plateaued at 500 nM (Figure 3A). Higher concentrations, up to 10 μM, did not further suppress metabolic activity, suggesting a plateau in the cellular response. This may reflect a combination of receptor occupancy, signalling adaptation, and/or other cellular mechanisms that limit the magnitude of the response at higher concentrations. SGT-24 also strongly recruits β-arrestin2 (EC_50_ ≈ 2.55 nM; Emax ≈ 206% relative to JWH-018), indicating that it can engage non-canonical signalling pathways associated with receptor desensitisation and internalisation [16]. Such signalling mechanisms may contribute to the observed suppression of metabolic activity, whereas receptor downregulation, signalling adaptation or other cellular compensatory mechanisms may limit further impairment and help preserve residual metabolic activity in cultured neural cells [32,41].

SGT-24 rapidly impaired mitochondrial function in rat cortical neurons and astrocytes, as evidenced by loss of ΔΨm (Figure 4A) and reduced ATP production (Figure 4B) within 120 min of exposure. These early bioenergetic deficits were followed by activation of distinct cell death pathways (Figure 5 and Figure 6), including caspase activation (Figure 7), indicating a rapid transition from metabolic dysfunction to cell death. Consistent with this cytotoxic profile, several SCRAs have been reported to induce acute toxicity across diverse cellular models. Short-term exposure (2 h) to cyclohexylphenol (CP) SCRAs at concentrations of 10–30 μM reduced viability in NG108-15 mouse neuroblastoma/rat glioma cells [33] and in cultured mouse forebrain neurons [33]. In murine Neuro-2a neuroblastoma cells, AB-FUBINACA, 5F-ADB-PINACA, and STS-135 exerted cytotoxic effects at 60 μM, while STS-135 showed marked toxicity at concentrations as low as 3 μM after only 1 h of exposure [34]. Similarly, human neurons, human astrocytes, and D384 astrocyte spheroids exhibited reduced viability following both short-term (3 h) and prolonged (48 h) exposure to low concentrations (1–5 μM) of MAM-2201 [37,38]. However, not all SCRAs induce overt cytotoxicity under comparable conditions. THJ-2201 and 5F-PB22 impaired neuronal differentiation at concentrations ≤ 1 μM without significantly affecting metabolic activity in NG108-15 cells [40]. Thus, SCRA-induced neurotoxicity can develop rapidly, within hours, and is strongly influenced by compound structure, concentration, exposure duration, and cellular context.

Although apoptotic pathways were activated in both, neurons and astrocytes, the two cell types exhibited distinct modes of cell death. Neurons predominantly underwent caspase-dependent apoptosis, as indicated by Annexin V/7-AAD staining (Figure 5) and characteristic apoptotic morphology (Figure 6), in line with earlier studies [33,37,42,43]. In contrast, astrocytes displayed a more heterogeneous phenotype, in which sustained caspase activation was accompanied by increased membrane permeability, consistent with progression towards secondary necrosis or other forms of cell death under prolonged stress (Figure 5 and Figure 6). This pattern is consistent with previous observations in glial cells exposed to neurotoxic insults [30,31,36,37,44,45,46].

Pharmacological profiling indicated that CB1 receptor activation is a major contributor to SGT-24-induced toxicity. Antagonism of CB1 with AM251 substantially restored mitochondrial function, improved ATP levels, and reduced cell death in both neurons and astrocytes (Figure 8 and Figure 9), consistent with a central role for CB1 receptors. Given the rapid mitochondrial impairment observed, involvement of mitochondrial CB1 (mtCB1) receptors is plausible [47,48]. Although SGT-24 exhibits off-target activity at other GPCRs, including chemokine, oxytocin, histamine H1, and α2B-adrenergic receptors, these interactions occur predominantly at micromolar concentrations [32], well above its nanomolar potency at CB1 receptors. In line with this, neither published data [32] nor our pharmacological inhibition experiments in astrocytes, in which we confirmed GPR55 expression, support a role for this receptor in SGT-24-induced toxicity.

Although CB1 activation appeared to account for most of the observed effects (Figure 8 and Figure 9), pharmacological inhibition also revealed contributions from TRPV1 and PPARγ, indicating that SGT-24 may engage multiple receptor-mediated pathways. TRPV1 is a stress-responsive, calcium-permeable channel implicated in nociception, neurogenic inflammation, synaptic plasticity, and neurotoxicity [49], whereas PPARγ is a nuclear receptor that regulates metabolism, energy homeostasis, differentiation, and inflammatory responses [20,50]. Cannabinoid actions may involve dual CB1/PPARγ signalling [21,50,51], although the underlying mechanisms remain unclear. THC can enhance PPARγ transcriptional activity and interact with its ligand-binding domain, supporting the possibility of dual receptor targeting [24,52,53]. Here, SGT-24-induced mitochondrial dysfunction and cellular stress were partly attenuated by CB1 or PPARγ antagonists (Figure 8), suggesting that PPARγ signalling may contribute to these effects either directly or downstream of CB1 activation, potentially through altered lipid signalling. Although PPARγ activation can exert protective effects [20,50,54], dysregulated PPARγ signalling may also contribute to oxidative stress and metabolic imbalance [10,51]. Moreover, the differential responses between neurons and astrocytes may reflect CB1 signalling bias, whereby different G-protein pathways are engaged depending on cell type, subcellular localisation, and receptor state [55]. Other SCRAs, including WIN55,212-2, CP55940, HU-331, and JWH-015, have also been reported to modulate PPARγ activity, with potential consequences for energy metabolism and neurotoxicity [20,21].

In addition to PPARγ, TRPV1 contributed selectively to the effects observed in neurons. Excessive TRPV1 activation can promote oxidative stress, mitochondrial dysfunction and intracellular Ca^2+^ overload [49,56,57], and comparable effects have been reported for several SCRAs, including XLR-11, UR-144, and AM1220 [58]. Thus, the present findings suggest that SGT-24 may engage multiple, as yet unknown, receptor-mediated pathways that converge on disruption of neuronal homeostasis and may contribute to neurotoxicity. Further studies are needed to clarify the receptor targets and downstream signalling pathways underlying the complex cellular effects of SGT-24.

## 4. Materials and Methods

### 4.1. Materials

Cumyl-PINACA (SGT-24; 1-pentyl-N-(2-phenylpropan-2-yl)-1H-indazole-3-carboxamide; purity > 98%) was kindly provided by the Slovenian National Forensic Laboratory (Ljubljana, Slovenia), while the CB1-selective antagonist/inverse agonist AM251, the GPR55 antagonist ML193, the TRPV1 antagonist AMG9810, and the PPARγ antagonist T0070907 were obtained from Tocris Bioscience (Bristol, UK). SGT-24 and all antagonists were dissolved in absolute ethanol and subsequently diluted in culture medium immediately before application to cells. Reagents and materials supplied with each kit are specified within the respective methodological descriptions.

### 4.2. Primary Cortical Neuron Culture

Primary neurons were prepared from the cerebral cortices of Wistar rat fetuses (E18-19) using a protocol approved by the Veterinary Administration of the Republic of Slovenia (No. U34401-3/2013/3), as previously described [46]. Cells were plated on vessels coated with poly-L-lysine (Sigma-Aldrich, Merck, St. Louis, MO, USA) and cultured in Neurobasal medium with 2% B27 Supplement, 0.5 mM GlutaMax, and 5 µg/mL gentamicin (all Gibco BRL, Scotland, UK). Cytosine arabinoside (2 µM, Sigma) was added on day 3. Half of the medium was replaced every 3–4 days with fresh medium without cytosine arabinoside. Experiments were conducted at 10–12 days in vitro.

### 4.3. Primary Cortical Astrocyte Culture

Primary astrocytes were prepared from the cerebral cortices of 2-day-old neonatal Wistar rats, following the protocol approved by the Veterinary Administration of the Republic of Slovenia (No. 34401-87/2008/3), as previously described [59]. Cells were cultured in DMEM/F12 (1:1) supplemented with 10% (vol/vol) FBS, 100 U/mL penicillin, and 100 mg/mL streptomycin (all from Gibco BRL, Scotland, UK), and maintained in a humidified atmosphere of 5% CO_2_ at 37 °C. After three overnight shakings, the monolayer cells were trypsinised, subcultured, and re-plated at an appropriate density in 6-well and 96-well culture plates (Nunc, Roskilde, Denmark) or on poly-L-lysine-coated coverslips.

### 4.4. Treatment

Cultured cells were incubated under serum-free conditions with SGT-24 (1 nM to 10 µM) or vehicle (0.001% *v*/*v* ethanol) for 24 h. In additional experiments, cells were treated with or without the selective CB1 antagonist/inverse agonist AM251, the GPR55 antagonist ML193, the TRPV1 antagonist AMG9810, or the PPARγ antagonist T0070907 (1 μM), together with 100 nM SGT-24 in cultured neurons or 500 nM SGT-24 in cultured astrocytes, for 1 to 24 h.

### 4.5. Quantitative Real-Time PCR

Total RNA (totRNA) was extracted from cultured neurons and astrocytes using the RNAqueous-Micro kit (Ambion, Life Technologies, Carlsbad, CA, USA). For reverse transcription, 10 μL of totRNA from each sample was utilized with the High-Capacity Reverse Transcription reagent (Applied Biosystems, Life Technologies, Carlsbad, CA, USA) following the manufacturer’s instructions. Real-time PCR reactions were performed for cannabinoid receptors CB1 and CB2, G protein-coupled receptor 55 (GPR55), transient receptor potential cation channel subfamily V member 1 (TRPV1), peroxisome proliferator-activator receptor gamma (PPARγ), and beta-actin. TaqMan Gene Expression Assays (Applied Biosystems) were employed for this purpose, with specific assay IDs (Rn02758689_s1, Rn01637601_m1, Rn03037213_s1, Rn00583117_m1, Rn00440945_m1, and Rn00667869_m1). The reactions, carried out in 10 μL volumes using Gene Expression Master Mix (Applied Biosystems), followed the manufacturer’s instructions and employed the universal amplification protocol on an ABI PRISM 7500 Fast instrument (Applied Biosystems). Cycle thresholds (Ct) for *Cnr1*, *Cnr2*, *Gpr55*, *Trpv1*, and *Pparg* target genes were normalized to the endogenous housekeeping gene *Actb*. Data were analyzed using the comparative Ct method ([delta][delta]Ct). The amplification efficiency of the target and the endogenous reference were approximately equal.

### 4.6. Western Blot Analysis

All reagents were purchased from Sigma-Aldrich, unless otherwise stated. Antibodies: anti-GPR55 (ab203663, Abcam, Cambridge, UK), anti-CB1 (ab23703, Abcam), anti-CB2 (ab3561, Abcam), anti-TRPV1 (ab6166, Abcam), anti-PPARγ (ab209350, Abcam), goat anti-rabbit and goat anti-mouse antibodies conjugated to horseradish peroxidase (Bio-Rad, Hercules, CA, USA).

Neuronal and astrocyte cultures were washed with ice-cold PBS and lysed in lysis buffer (20 mM Hepes-KOH (H3375), pH 7.9, 125 mM NaCl (31434), 1 mM EDTA (E6758), 1% Igepal CA-360 (I8896), 10 mM sodium pyrophosphate (P8010), 5 mM sodium fluoride (S6776), 5 mM β-glycerol phosphate (50020), 0.2 mM sodium orthovanadate (450243), 1 mM phenylmethylsulfonyl fluoride (PMSF, P7626) and 1 mM protease inhibitor cocktail (P8340)). The lysates were centrifuged to yield the total protein extract and the protein concentration was measured. Equal amounts of samples (15 μg) were denatured and applied on 12% (*w*/*v*) acrylamide gels to be separated by standard sodium dodecyl sulfate polyacrylamide gel electrophoresis (SDS-PAGE) and blotted onto PVDF membrane (Immobilon-P; Merck-Millipore, Darmstadt, Germany). The membranes were blocked with 3% BSA in TBST for 1 h at room temperature (RT), incubated with primary antibody for 1.5 h at RT or overnight at 4 °C, and subsequently with horseradish peroxidase (HRP)-conjugated secondary antibody for 1.5 h at room temperature. The luminescence signal was visualized using a Fusion FX imager (Vilber, Marne-la-Vallée, France).

### 4.7. Cellular Metabolic Activity and Death Analysis

- Metabolic activity: Cellular metabolic activity was assessed by quantifying the reduction in the AlamarBlue^®^ redox indicator resazurin (Molecular Probes, Eugene, OR, USA) according to the manufacturer’s instructions. Fluorescence was measured at excitation and emission wavelengths of 540 nm and 595 nm, respectively, using a Synergy HT microplate reader (BioTek, Winooski, VT, USA). Data, expressed as relative fluorescence units (RFU), were normalised to protein concentration, determined using the Bradford method [60] with the Bio-Rad Protein Assay (Hercules, CA, USA).
- Flow cytometry detection of cell death: The cells were stained simultaneously with Annexin V-FITC (Annexin V) and 7-Aminoactinomycin D (7-AAD), using the staining kit from Beckman Coulter (Brea, CA, USA). Briefly, cells were centrifuged at 500 rcf at 4 °C for 5 min, washed with cold PBS, and resuspended in 100 µL cold 1× binding buffer. Cells were then stained with 10 µL Annexin V and 20 µL 7-AAD solution. After a 15-min incubation in the dark at 4 °C, 400 µL of 1× binding buffer was added. Data acquisition was performed using a Quanta SC MPL flow cytometer (Beckman Coulter). Each measurement contained 10,000 counted cells. An Electronic Volume (EV) vs. Side Scatter (SS) plot was used as a gating layout to isolate intact cells and remove small debris. Annexin V was detected using a filter at a wavelength of 525 nm, and for the detection of 7-AAD, a filter of 670 nm was used. For cell death analysis, early apoptotic cells were defined as Annexin V positive and 7-AAD negative, while late-stage apoptotic and secondary necrotic cells were defined as positive for both Annexin V and 7-AAD.

### 4.8. Change in Mitochondrial Membrane Potential (ΔΨm)

Cells were subjected to various treatments and then incubated with the JC-1 probe (Cayman Chemical Company, Ann Arbor, MI, USA) at a concentration of 5 μg/mL at 37 °C for 15 min. Subsequently, cells were washed twice with assay buffer, and fluorescence was measured using a Synergy HT microplate reader (BioTek, Winooski, VT, USA). The ratio of fluorescence intensity between JC-1 aggregates and monomers was used as an indicator of mitochondrial membrane potential.

### 4.9. ATP Measurement

Intracellular ATP concentration was determined using the ATPlite 1-step assay system (PerkinElmer, Boston, MA, USA), which generates a luminescence signal proportional to the amount of ATP present. ATP content was calculated using a standard ATP curve (1 pM–1 µM) and normalised to protein concentration determined by the Bradford method [60].

### 4.10. Detection of Caspase Activity

After exposure to cannabinoids, cells were harvested and solubilised in cell culture lysis buffer. The activities of caspase-8, caspase-9, and caspases-3/7 were determined by measuring the luminescent substrates using the Caspase-Glo 8, Caspase-Glo 9, and Caspase-Glo 3/7 assays (Promega, Madison, WI, USA).

### 4.11. Immunocytochemistry

Cells cultured on coverslips were rinsed with PBS, fixed in 4% paraformaldehyde (Sigma) for 15 min, and permeabilised with 0.1% Triton-X-100 for 5 min at room temperature. Coverslips were incubated overnight at 4 °C with rabbit anti-β III tubulin (1:500, ab18207, Abcam) and/or mouse anti-glial fibrillary acidic protein (GFAP) (1:500, MAB360, Millipore, Burlington, MA, USA), washed and incubated with the corresponding Alexa Fluor 488- and 546-conjugated secondary antibodies (1:500, Molecular Probes, Eugene, OR, USA) for 1 h at 37 °C. Nuclei were stained with Hoechst (2 µg/mL, Molecular Probes) for 10 min at 37 °C. After washing, coverslips were mounted in ProLong Diamond (Molecular Probes) and observed under a fluorescence microscope (Olympus, Tokyo, Japan).

### 4.12. Data Analysis

Statistical analysis of the data and graphical presentation of the results were performed using GraphPad Prism, version 10.4.1 (GraphPad Software Inc., San Diego, CA, USA; www.graphpad.com). Data are presented as mean ± SEM from five independent experiments (*n* = 5), with each experiment conducted in triplicate. Statistical comparisons were performed using one-way analysis of variance (ANOVA) followed by Tukey’s or Dunnett’s post hoc test, as appropriate, for multiple-group comparisons, or an independent- samples *t*-test for comparisons between two groups. The specific statistical test used for each experiment is indicated in the corresponding figure legend. A *p* value < 0.05 was considered statistically significant. The graphical abstract was created using BioRender.com (BioRender, Toronto, ON, Canada; accessed July 2026).

## 5. Conclusions

SGT-24 induces potent, cell-type-dependent neuroglial toxicity associated with mitochondrial dysfunction and distinct patterns of cell death, suggesting the involvement of both cannabinoid-receptor-dependent and non-cannabinoid signalling mechanisms. Signalling via the CB1 receptor appears to play a predominant role in SGT-24-induced cellular toxicity, with TRPV1- and PPARγ-related pathways possibly also contributing. The nanomolar potency of SGT-24 underscores the toxicological risk posed by high-potency SCRAs. Further studies using complementary pharmacological and in vivo approaches are warranted to elucidate the receptor mechanisms underlying SGT-24 toxicity and their relevance to SCRA intoxication.

## Data Availability

The data that support the findings of this study are available from the corresponding author upon request.

## Supplementary Materials

The following supporting information can be downloaded at: https://www.mdpi.com/article/10.3390/ph19091436/s1, Figure S1. Effects of receptor antagonists alone on metabolic activity and mitochondrial function in cultured rat cortical neurons and astrocytes (antagonist-only controls); Figure S2. Effects of receptor antagonists alone on caspase activity in cultured rat cortical neurons and astrocytes.

## Abbreviations

The following abbreviations are used in this manuscript:

ATP	Adenosine triphosphate
CB1	Cannabinoid type 1 receptor
CB2	Cannabinoid type 2 receptor
CBRs	Canonical cannabinoid receptors
EC_50_	Half-maximal effective concentration
GPR55	G protein-coupled receptor 55
IC_50_	Half-maximal inhibitory concentration
PPARγ	Peroxisome proliferator-activated receptor gamma
RFU	Relative fluorescence units
SCRAs	Synthetic cannabinoid receptor agonists
SGT-24	Cumyl-PINACA, 1-pentyl-N-(2-phenylpropan-2-yl)-1H-indazole-3-carboxamide
THC	Δ^9^-tetrahydrocannabinol
TrpV1	Transient receptor potential cation channel subfamily V member 1
ΔΨm	Mitochondrial membrane potential
