# Supplementary material for: Neurotoxicity of Synthetic Cannabinoid Receptor Agonist Cumyl-PINACA: An In Vitro Study on Rat Cortical Neurons and Astrocytes
Source: Pharmaceuticals (Basel). 2026 Sep 10;19(9):1436. doi: 10.3390/ph19091436 (PMC13610142; doi:10.3390/ph19091436)
Supplement: Supplementary file 1 [file pharmaceuticals-19-01436-s001.zip › pharmaceuticals-4467533-supplementary.pdf]

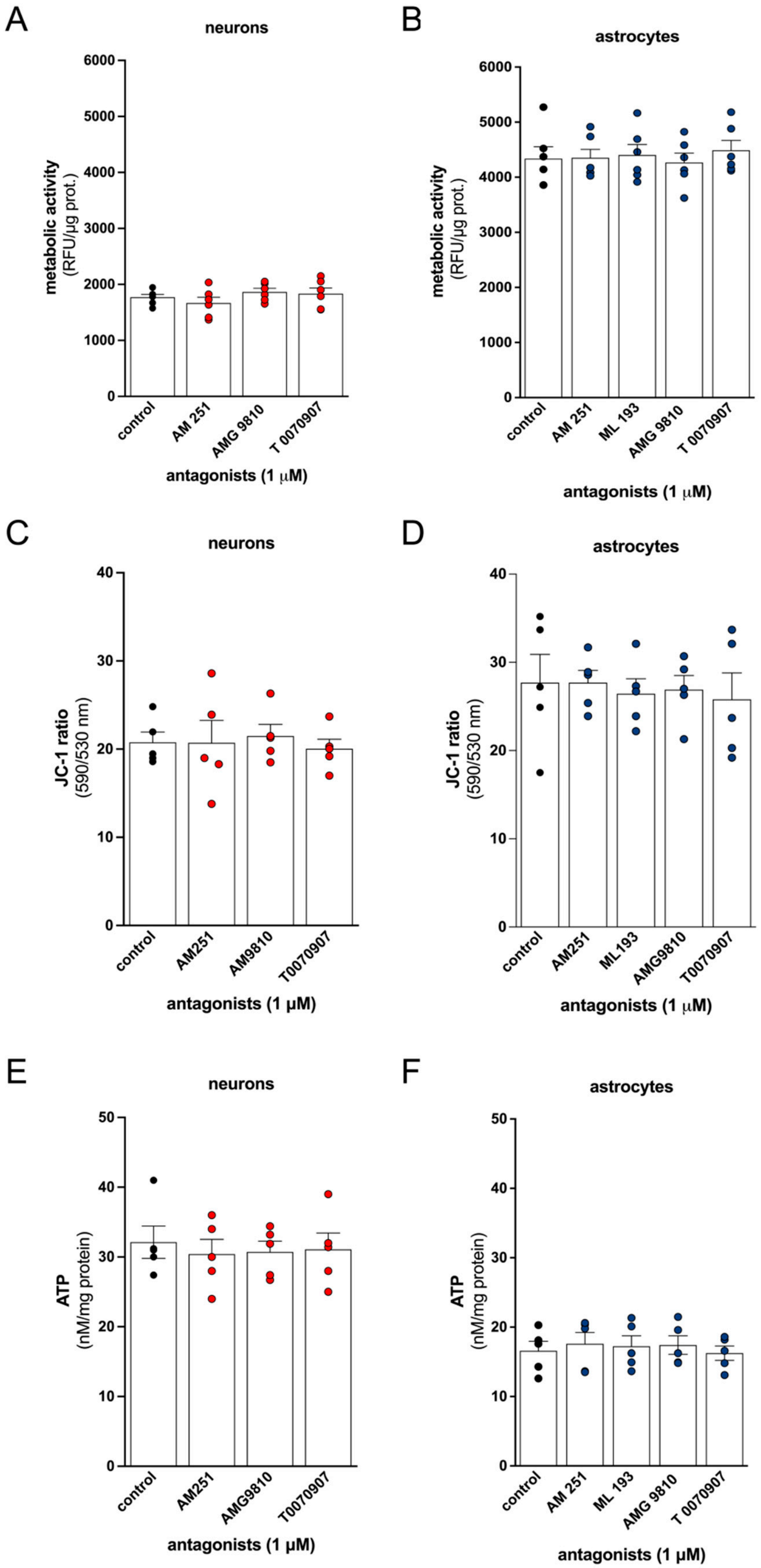

Figure S1. Effects of receptor antagonists alone on metabolic activity and mitochondrial function in cultured rat cortical neurons and astrocytes (antagonist-only controls). Neurons

were incubated for 24 h with selective antagonists of CB1 (AM251), TRPV1 (AMG 9810), or PPARγ (T0070907) (1 μM), whereas astrocytes were incubated for 24 h with selective antagonists of CB1 (AM251), GPR55 (ML193), TRPV1 (AMG 9810), or PPARγ (T0070907) (1 μM). (**A**), (**B**) Cellular metabolic activity was assessed using the AlamarBlue® assay. Data are shown as relative fluorescence units (RFU), normalised to protein content, and presented as mean ± SEM (n = 5). Statistical analysis was performed using one-way ANOVA followed by Dunnett's post hoc test. (**C**), (**D**) Mitochondrial membrane potential (ΔΨm) was assessed using the JC-1 dye. Data are presented as the JC-1 fluorescence ratio (mean ± SEM, n = 5). Statistical analysis was performed using one-way ANOVA followed by Dunnett's post hoc test. (**E**), (**F**) Cellular ATP content was determined using the ATPlite 1-step assay system and normalised to total protein concentration. Results are presented as mean ± SEM (n = 5). Statistical analysis was performed using one-way ANOVA followed by Dunnett's post hoc test.

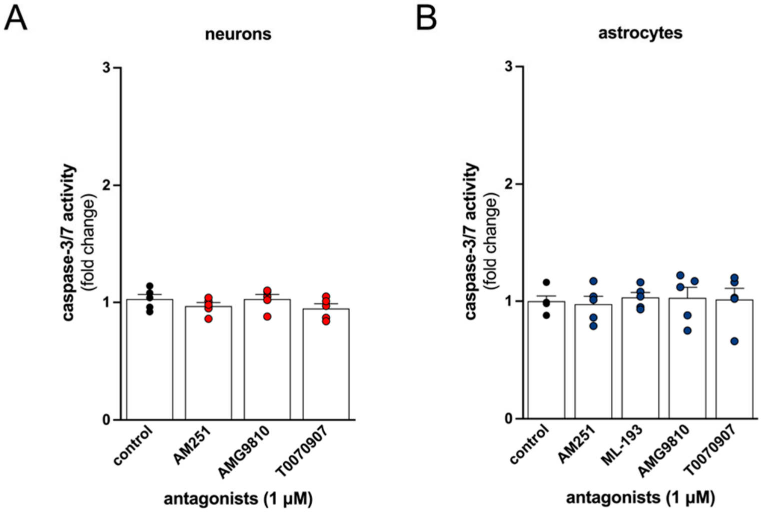

Figure S2. Effects of receptor antagonists alone on caspase activity in cultured rat cortical neurons and astrocytes (antagonist-only controls). Neurons were incubated for 24 h with selective antagonists of CB1 (AM251), TRPV1 (AMG 9810), or PPARγ (T0070907) (1 μM), whereas astrocytes were incubated for 24 h with selective antagonists of CB1 (AM251), GPR55 (ML193), TRPV1 (AMG 9810), or PPARγ (T0070907) (1 μM). (A), (B) Caspase activity was evaluated using Caspase-Glo 3/7 assays; data were normalised to the mean value of the control and are presented as mean ± SEM (n = 5). Statistical analysis was performed using one-way ANOVA with Dunnett's post hoc test.
